# Wasteful Azo Dyes as a Source of Biologically Active Building Blocks

**DOI:** 10.3389/fbioe.2021.672436

**Published:** 2021-06-15

**Authors:** Ana Fernandes, Bruna Pinto, Lorenzo Bonardo, Beatriz Royo, M. Paula Robalo, Lígia O. Martins

**Affiliations:** ^1^Instituto de Tecnologia Química e Biológica António Xavier, Universidade Nova de Lisboa, Oeiras, Portugal; ^2^Área Departamental de Engenharia Química, Instituto Superior de Engenharia de Lisboa (ISEL), Instituto Politécnico de Lisboa, Lisbon, Portugal; ^3^Centro de Química Estrutural, Complexo I, Instituto Superior Técnico, Universidade de Lisboa, Lisbon, Portugal

**Keywords:** laccases, aromatic amines, whole-cell catalysis, phenazines, phenoxazinones, azoreductase

## Abstract

In this work, an environment-friendly enzymatic strategy was developed for the valorisation of dye-containing wastewaters. We set up biocatalytic processes for the conversion of azo dyes representative of the main classes used in the textile industry into valuable aromatic compounds: aromatic amines, phenoxazinones, phenazines, and naphthoquinones. First, purified preparations of PpAzoR azoreductase efficiently reduced mordant, acid, reactive, and direct azo dyes into aromatic amines, and CotA-laccase oxidised these compounds into phenazines, phenoxazinones, and naphthoquinones. Second, whole cells containing the overproduced enzymes were utilised in the two-step enzymatic conversion of the model mordant black 9 dye into sodium 2-amino-3-oxo-3*H*-phenoxazine-8-sulphonate, allowing to overcome the drawbacks associated with the use of expensive purified enzymes, co-factors, or exquisite reaction conditions. Third, cells immobilised in sodium alginate allowed recycling the biocatalysts and achieving very good to excellent final phenoxazine product yields (up to 80%) in water and with less impurities in the final reaction mixtures. Finally, one-pot systems using recycled immobilised cells co-producing both enzymes resulted in the highest phenoxazinone yields (90%) through the sequential use of static and stirring conditions, controlling the oxygenation of reaction mixtures and the successive activity of azoreductase (anaerobic) and laccase (aerobic).

## Introduction

More than 100,000 different recalcitrant synthetic dyes are currently used in the textile, food, paper, printing, leather, and cosmetics industries (Carmen and Daniela, 2012). It is estimated that around 10^5^ tonnes of dyes and dyestuff are released into the environment in the 200 billion litres of wastewaters, which result annually from the aforementioned industrial activities (Kant, 2012). Wastewaters containing dyes, from which 70% are azo dyes with one or more azo bonds (R_1_–N-N–R_2_), in addition to aesthetic problems, represent a liability since many azo dyes as well as their breakdown products are toxic and potentially mutagenic to living organisms (Rawat et al., 2016; Hassaan and Nemr, 2017).

The vast majority of dye-containing wastewater treatments rely on the use of physico-chemical methods, including precipitation, coagulation, and filtration (Pereira and Alves, 2012; Vikrant et al., 2018). In spite of their recognised efficiency in decolourisation processes, they rely on the use of hazardous additives, produce large amounts of sludge, and are frequently not skilful for full wastewater detoxification. Biological treatment technologies are eco-friendly and cost-effective alternatives (Rai et al., 2005; Saratale et al., 2011; Khan et al., 2013). In particular, enzymatic processes are particularly attractive considering that biological decolourisation is assigned to the action of a limited number of enzymes: azoreductases, laccases, and peroxidases (Rodriguez-Couto, 2009; Mendes et al., 2015; Singh et al., 2015). Enzymatic processes are sustainable, eco-friendly, and particularly attractive considering their specificity for dye degradation while keeping intact valuable dyeing additives or fibres that can potentially be re-used, resulting in significant reductions in water consumption. At present, treatment of dye-containing wastewaters is mostly focused in their remediation; however, the implementation of appropriate approaches has potential to couple the degradation of dyes to their conversion into valuable chemicals. This is in line with the principles of circular economy emphasising the urgency to design out waste and pollution, keep products, and materials in use and regenerate natural systems (Geissdoerfer et al., 2017; Lange et al., 2021).

In a previous work, we showed that the coupled action of PpAzoR azoreductase from *Pseudomonas putida* MET94 and CotA-laccase from *Bacillus subtilis* resulted in the decolourisation as well as in the detoxification of a large array of structurally diverse azo dyes and model wastewaters (Mendes et al., 2011a). Herein, we report the coupled action of these enzymes in the valorisation of azo dyes commonly present in wastewaters. Azoreductases are highly effective in decolourising azo dyes yielding aromatic amines, building blocks in different industries, including agrochemical, fine chemical, and pharmaceutical. CotA-laccase oxidises a wide range of aromatic amines with different substitution patterns (*ortho*-phenylenediamines, substituted *para*-phenylenediamines, and *ortho*-amino-phenols, among others) that are precursors of dimeric and trimeric dyes as well as of substituted heterocyclic frameworks (phenazine, phenoxazinone, and carbazole derivatives), multifunctional and versatile building blocks widely distributed in a vast array of biologically active compounds (Martins et al., 2020). The five dyes used in this study, namely, mordant black 9 (MB9), mordant black 3 (MB3), acid red 266 (AR266), reactive yellow 145 (RY145), and direct red 80 (DR80), were chosen as they represent the most common classes of azo dyes used in the textile industry, for example, in cotton and wool-dyeing processes (Carmen and Daniela, 2012), and also because we had previous results showing that the sequential treatment with PpAzoR and CotA-laccase results in maximal decolourisation and detoxification levels (Mendes et al., 2011a).

In this work, we have identified, using NMR spectroscopy, the aromatic amines resulting from azo dye degradation by the PpAzoR azoreductase, and the heterocyclic products (phenazines, phenoxazinones, and naphthoquinones) that resulted from the oxidation of these aromatic amines with CotA-laccase. We have optimised using the MB9 azo dye, two-step and one-pot bioprocesses using free and immobilised *Escherichia coli* cells that have overproduced the enzymes of interest, resulting in reactions performed in water and at mild conditions of temperature, which led to 90% phenoxazine conversion yields.

## Materials and Methods

### Chemicals

All chemicals and solvents were obtained from commercial suppliers and were used without further purification. The dyes used in this study were as follows: MB9 (λ_max_ = 550 nm; purity of 48%), MB3 (λ_max_ = 550 nm; purity of 40%), AR266 (λ_max_ = 470 nm; purity of 30%), RY145 (λ_max_ = 420 nm; purity of 50%), and DR80 (λ_max_ = 530 nm; purity of 50%). MB9 was purchased from DyStar Textilfarben (Frankfurt, Germany) and MB3 from Honeywell Fluka (Bucharest, Romania); and AR266, RY145, and DR80 were purchased from Town End (Leeds, United Kingdom).

### Bacterial Strains and Plasmids

*Escherichia coli* strain DH5α (Novagen, Madison, WI, United States) was used for routine propagation and amplification of plasmid constructs; and *E. coli* Tuner (DE3, Novagen), *E. coli* KRX (Promega, Madison, WI, United States), and *E. coli* BL21 star (DE3, Novagen) were tested as host of plasmids for gene expression. Three plasmids were used: pLOM10, coding for CotA-laccase from *B. subtilis* (Martins et al., 2002); pVB-8, coding for the evolved variant 2A1-Y179H of *P. putida* MET94 PpAzoR (Brissos et al., 2014); and pAIF-2, a pET-Duet-1 construct, coding for both wild-type PpAzoR and CotA-laccase genes (Mendes et al., 2011a).

### Preparation of Purified Enzymes and Whole-Cell Systems

*Escherichia coli* recombinant cells overproducing the enzymes of interest were cultivated in Luria–Bertani (LB) media supplemented with 100 μg ml^–1^ of ampicillin following previously described procedures (Durao et al., 2008; Mendes et al., 2011b). The cells were harvested by centrifugation (8,000 × *g*, 10 min, 4°C) and washed with 0.9% (w/v) NaCl.

(i) For reactions using purified enzymes, cells were disrupted using a French press, cell debris were removed by centrifugation (18,000 × *g*, 2 h, 4°C), and enzymes were purified from supernatants using well-established chromatographic methods (Bento et al., 2005; Mendes et al., 2011b). CotA-laccase activity was determined at 37°C using 0.1 mM of ABTS as substrate (ε_420_ = 36,000 M^–1^ cm^–1^) in 0.1 M of sodium acetate buffer, pH 4.3. The activity of PpAzoR azoreductase was measured in 0.1 M of sodium phosphate buffer, pH 7, using 0.1 mM of anthraquinone-2-sulphonate (AQS) and 0.25 mM of β-nicotinamide adenine dinucleotide phosphate (NADPH; ε_340_ = 6,200 M^–1^ cm^–1^) at 42°C. One unit (U) of enzymatic activity was defined as the amount of enzyme required to convert 1 μmol of substrate per min. Aliquots of purified enzymes were stored at –20°C prior to use. The protein concentration was determined using the Bradford assay with bovine serum albumin as the standard.
(ii) For reactions with free and immobilised whole cells, *E. coli* cells were suspended in distilled water, the optical density was measured at 600 nm (OD_600_), aliquots of cell suspensions containing around 300-mg dry cell weight (DCW) were centrifuged, and pellets stored at –20°C prior to use. A calibration curve of OD_600_ versus DCW (mg ml^–1^) after drying cell suspensions (at different OD_600_) at 100°C until no variation in weight was previously performed. Immobilisation was performed after suspending cell pellets (300- or 600-mg DCW) in 2 ml of a 2% sodium alginate solution, followed by dropwise addition to a 0.15 M of CaCl_2_ solution under continuous stirring. The spheres were left to solidify for 90 min, then were washed with distilled water, and stored at 4°C until use.

### Two-Step Enzymatic Bioprocess Using Enzymes

The first step of bioconversion of dyes using purified PpAzoR azoreductase was performed in serum bottles (20 ml) containing 2 mM of β-nicotinamide adenine dinucleotide hydrogen (NADH) and 0.8–1.0 mM of azo dyes (MB9, MB3, AR266, RY145, and DR80) in 0.1 M of sodium phosphate buffer, pH 7, made anaerobic by nitrogen bubbling followed by sealing with rubber stoppers. The 10-ml mixtures were incubated at 30°C, and reactions were started by adding 5 U ml^–1^ of an anoxic preparation of purified enzyme. Decolourisation was monitored at the maximum wavelength of each dye. When decolourisation was ≥85%, reaction mixtures were transferred to a 50-ml Erlenmeyer flask, 1 U ml^–1^ of CotA-laccase was added, and these were shaken at 180 rpm at 37°C for 24 h.

### Two-Step Enzymatic Bioprocess Using Free and Immobilised Whole Cells

The first PpAzoR catalysed step was performed in 5 ml of water or 20 mM of sodium phosphate buffer (pH 7) at 30°C in the presence of free or immobilised cells (60 or 120 mg DCW ml^–1^). Reactions were started by the addition of MB9 to a final concentration of 0.96 mM. Decolourisation was monitored at 550 nm (λ_max_ for MB9). After decolourisation levels reached values higher than 85% (∼24 h of reaction), mixtures were shaken at 180 rpm, at 37°C, for an additional period of 24 h. Recycled biocatalysts were tested for the first enzymatic step, as follows: after 24 h of reaction, 5 ml of 0.96 mM of MB9 was added to the reaction mixture. This procedure was repeated four times in 24-h periods up to a maximum of 96 h. After this time, the second step with CotA-laccase was performed as described above. The products of reactions after the first and second enzymatic steps were quantified by high-performance liquid chromatography (HPLC) using a calibration curve performed with an isolated final compound (please see below). Each experience was performed at least five times, and standard deviations were lower than 15%.

### Product Characterisation

UV–visible spectra of substrates and reaction mixtures were obtained on a Nicolet Evolution 300 spectrophotometer (Thermo Fisher Scientific, Waltham, MA, United States). Decolourisation was assessed by measuring the absorbance of reaction mixtures at the wavelength of maximal absorbance for each dye tested [decolourisation (%) = (Abs initial-Abs final)/Abs final × 100]. For the identification of products, reaction mixtures were lyophilised, suspended in appropriate deuterated solvent, and analysed by NMR. ^1^H- and ^13^C-NMR spectra were obtained at room temperature with an Advance Bruker 400 MHz spectrometer (Billerica, MA, United States) in CD_3_OD-*d*_4_ or D_2_O solvents (see Supplementary Table 1). The chemical shifts are reported in part per million (ppm) using the solvent signal as internal reference. The final product of MB9 conversion, after the sequential action of PpAzoR and CotA-laccase, was separated and collected by HPLC, and its molecular extinction coefficient was determined in the concentration range of 0.05–0.8 mM at 430 nm using a Synergy 2, BioTek (Winooski, VT, United States) microplate reader. For the quantification of products of reactions using MB9, samples of reaction were centrifuged and analysed by HPLC on a Waters Alliance 2695 equipped with a Waters photodiode array detector. The separations were performed in a Purospher STAR RP-18e column (250 mm × 4 mm), 5-μm particle size (Merck, KGaA, Gernsheim, Germany). The injection volume was 60 μl, and the flow rate was 0.8 ml min^–1^ at a column oven temperature of 40°C. Eluent A was 0.1 M of ammonium acetate pH 6.7, and eluent B was a mixture of methanol:acetonitrile (70:30, v/v). The following gradient was used for products separation: 0–2 min, isocratic elution of 100% eluent A; 2–16 min, linear gradient from 100 to 60% of eluent A; 16–24 min, linear gradient from 60 to 45% of eluent A; 24–28 min, linear gradient from 45 to 20% of eluent A; 28–32 min, isocratic elution of 20% eluent A; 32–30 min, linear gradient from 20 to 100% of eluent A; and 33–43 min, equilibrium to the initial conditions of the following injection.

## Results and Discussion

### Two-Step Bioconversion of Dyes Using Purified Enzymes

**FIGURE 1:**
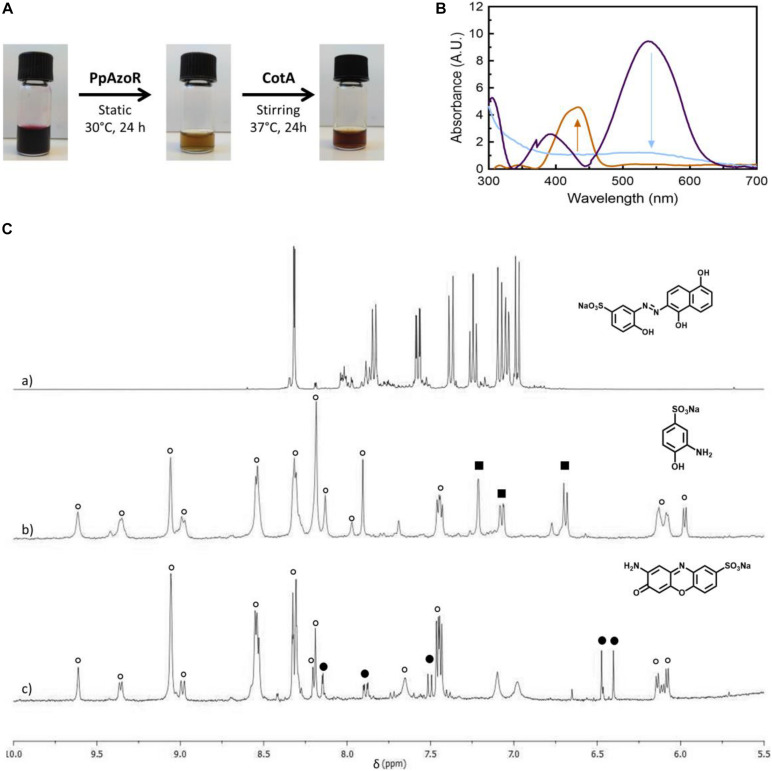
**(A)** Products of the MB9 bioconversion upon the sequential reaction with purified PpAzoR azoreductase and CotA-laccase, under anaerobic and aerobic conditions, respectively. **(B)** UV–Vis spectra of the initial reaction mixture (purple) and after reaction with PpAzoR azoreductase (light blue) and CotA-laccase (light brown). **(C)** ^1^H-NMR spectra (aromatic region) of MB9 **(a)** and of the products of reaction after addition of PpAzoR azoreductase **(b)** and CotA-laccase **(c)**. Resonances due to NAD^+^ and other intermediates resulting from NADH/NAD^+^ degradation (open circles), SAHBS (filled squares), and compound 7 (filled circles) are labelled.

The reactions of PpAzoR azoreductase, with the five azo dyes, were performed under anaerobic conditions and yielded decolourisation levels above 85% after 24 h of reaction as assessed by UV–Vis at the λ_max_ of each dye (Table 1). The final reaction mixtures were analysed by ^1^H-NMR spectroscopy (in CD_3_OD-*d*_4_; Figure 1 and Supplementary Figures 1–4), which indicated the complete disappearance of the initial dyes and the presence of aromatic amines as well as NAD^+^, the oxidation product of NADH and other compounds derived from the NADH/NAD^+^ degradation. In the reduction of the MB9 by the PpAzoR one aromatic amine, SAHBS was detected in the ^1^H-NMR spectrum (Figure 1C,b), similarly to the other dyes where only one of the two expected amines was identified (Supplementary Table 2), except in the case of DR80 dye, where two of the three expected amines were identified (Table 1). SAHNS, 2 (Supplementary Figure 1C), SDAHNS, 3 (Supplementary Figure 2C), and SANTS, 4 (Supplementary Figure 3C) are products of the reaction with MB3, AR266, and RY145, respectively; and SABS, 5, and SDBS, 6 (Supplementary Figure 4C) are from the reaction with DR80. The absence of some expected amines in the reaction mixtures is attributed to their autoxidation, upon exposure to oxygen, leading to the production of unstable forms and further involvement in oligomeric or polymeric reactions, as previously reported (Kudlich et al., 1999; Pinheiro et al., 2004; Carvalho et al., 2008).

**TABLE 1:**
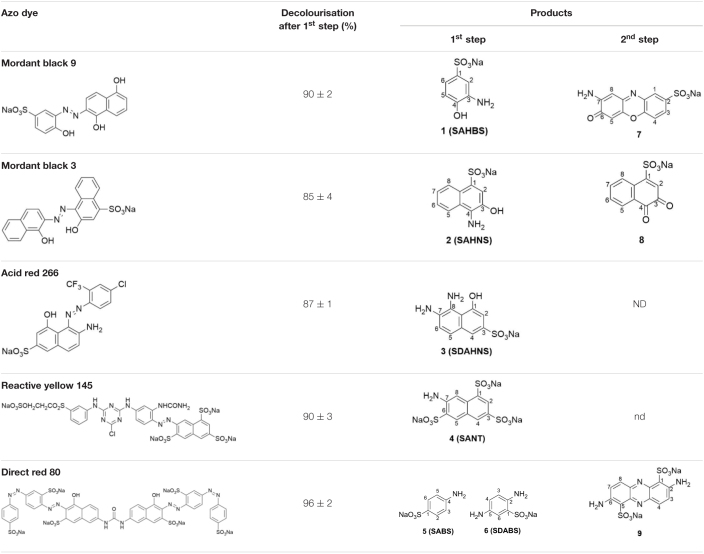
Dyes used in this study and products obtained after reaction with the PpAzoR azoreductase (1^st^ step) and CotA-laccase (2^nd^ step), as identified by NMR spectroscopy.

*SAHBS, sodium 3-amino-4-hydroxybenzene-1-sulphonate; SAHNS, sodium 4-amino-3-hydroxynaphthalene-1-sulphonate; SDAHNS, sodium 5,6-diamino-4-hydroxynaphthalene-2-sulphonate; SANTS, sodium 7-aminonaphthalene-1,3,6-trisulphonate; SABS, sodium 4-aminobenzene-1-sulphonate; SDBS, sodium 2,5-diaminobenzene sulphonate; ND, not determined; nd, not detected.*

The second step of the biotransformation was initiated upon addition of CotA-laccase and set-up of aerobic conditions (Martins et al., 2002). The oxidation of SAHBS (1), the PpAzoR product from the MB9 dye, resulted in the formation of the coloured sodium 2-amino-3-oxo-3*H*-phenoxazine-8-sulphonate (7) (Forte et al., 2010). The oxidation of compound 2, the PpAzoR product from MB3, led to the formation of *ortho*-naphthoquinone (8) (Sousa, 2015). Considering the enzymatic pathway proposed for the oxidation of *ortho*-aminophenol derivatives mediated by CotA-laccase, the *ortho*-quinone core can be obtained through a hydrolysis process of the *ortho*-quinoneimine intermediate formed during CotA-laccase oxidation (Sousa et al., 2014). The CotA-laccase oxidation of the SDAHNS (3), obtained from AR266, originates a complex mixture of products, resulting from putative reoxidation, polymerisation, or hydrolysis processes, which impaired their full characterisation. For RY145, the analysis of the mixture after the second enzymatic oxidative step showed that amine (4) is not a substrate of CotA-laccase probably due to the presence of the three electron-withdrawing sulphonate groups, thereby decreasing the electron density in the naphthalenic structure and making the amine group less favourable to oxidation (Sousa et al., 2013). The mixture obtained by CotA-laccase oxidation of amines SABS (5) and SDBS (6) reveals the presence of a coloured phenazine (9), obtained from the self-coupling reaction of SDBS (Sousa et al., 2014), while SABS (5) due to the presence of the electron-deficient sulphonate group in the *para* position is not a CotA-laccase substrate (Sousa et al., 2013). Overall, our results show that the sequential use of PpAzoR and CotA results in the conversion of all azo dyes into structurally different building blocks, aromatic amines, naphthoquinones, phenoxazinones, and phenazines. These are biological active motifs of antibiotics (McDonald et al., 1999), antitumor agents (Corona et al., 2009; Vairavelu et al., 2014), and pesticides (Piro et al., 2013). They are also useful precursors for the manufacturing of pharmaceuticals (Cholo et al., 2012), fine chemicals (Forte et al., 2010; Morel and Christie, 2011), and biosensors (Piro et al., 2013). Furthermore, synthetic chemical approaches for the synthesis of these heteroaromatic compounds use stoichiometric amounts of chemicals, strong acids, and high temperatures and result in low production yields (Shruti et al., 2018). In order to set up not only environment-friendly but also cost-effective processes, we performed dye conversion assays using free and immobilised whole-cell systems that overproduced the enzymes of interest.

### Set-Up of Free and Immobilised Whole-Cell Systems

**FIGURE 2:**
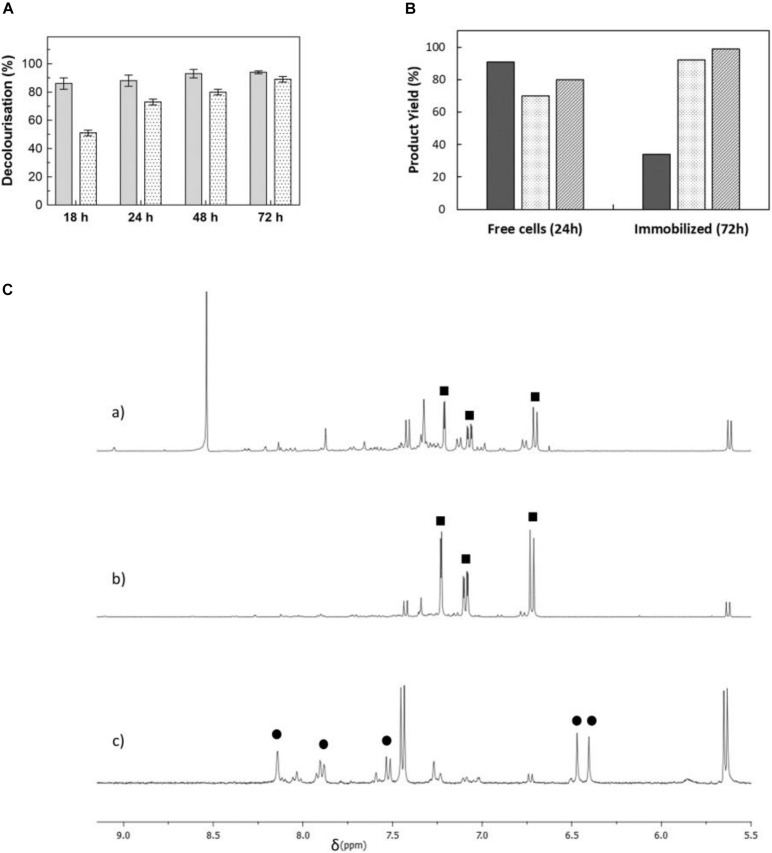
**(A)** Time-course decolourisation of MB9 using free (light grey bars) and immobilised (dotted bars) cells containing PpAzoR azoreductase (60 mg DCW ml^–1^) at 30°C in static conditions. **(B)** Yields of SAHBS in processes using free and immobilised cells of *Escherichia coli* Tuner (light black), BL21 star (light grey), and KRX cells (dark grey). **(C)** ^1^H-NMR spectra (aromatic region) of reaction mixtures containing MB9 with **(a)** free or **(b)** immobilised *E. coli* KRX cells overproducing PpAzoR in static conditions at 30°C and **(c)** after addition of immobilised *E. coli* KRX cells overproducing CotA-laccase under stirring conditions at 37°C. Resonances due to SAHBS (filled squares) and compound 7 (filled circles) are labelled.

Whole-cell bioprocesses are very advantageous since they reduce operational costs associated with enzyme purification and the supply of expensive co-factors such as NAD(P)H, which is recycled by the metabolism of resting cells (Carvalho, 2016; Lin and Tao, 2017). Moreover, processes with whole cells (i.e., resting cells) are very advantageous since cells efficiently take out oxygen from the media and allow the anaerobic PpAzoR to decolourise azo dyes while avoiding the need of using flasks sealed with rubber stoppers and made anaerobic by argon bubbling. Therefore, time-course decolourisation assays of the model dye MB9 were performed using different *E. coli* strains overproducing PpAzoR variant 2A1-Y179H, which shows fourfold higher activity than the wild-type enzyme (Brissos et al., 2014). Whole-cell reactions resulted in decolourisation levels close to 95% with 70–91% product yields (0.66–0.87 mM of SAHBS; Figures 2A,B) after 24 h of reaction. In general, it was observed that the levels of decolourisation were higher than product yields most likely related to adsorption of dyes to cell surfaces (Prigione et al., 2008). The ^1^H-NMR analysis of the reaction mixtures showed only resonances attributed to SAHBS (Figures 2C,a), in contrast to the reactions using purified enzymes where NADPH was added to the reaction mixtures (see Figures 1C,b), but also the presence of other signals, which are due to intracellular metabolites derived from cells. Next, we have performed reactions in alginate-immobilised cells, considering that immobilisation eases the recycling of biocatalysts, which is critical for the implementation of cost-effective processes (Devi et al., 2017). Decolourisation with immobilised cells was slower (∼90% decolourisation only after 72 h; Figure 2B), most likely due to the presence of the alginate matrix that restricts diffusion of substrate through the beads. However, reactions with immobilised cells displayed a cleaner ^1^H-NMR spectrum indicating that cell encapsulation prevented leakage of undesirable intracellular metabolites (Figures 2C,b). *E. coli* KRX cells were selected for further studies considering their superior performance (Figure 2B and Supplementary Figure 5). The amount of cells in alginate beads was increased from 60 to 120 mg DCW ml^–1^, and buffer was replaced by water to increase the cost-effectiveness and sustainability of the bioprocess, and after 24 h, a significant increase in decolourisation of MB9 (89%) was achieved (Supplementary Figure 6). In these conditions, the conversion yields of MB9 to SAHBS reached 74% (Table 2). The addition of CotA-laccase resulted in the full conversion (99%) of the amine to the phenoxazinone (7) (Figures 2C,c and Table 2). Next, we tested a recycling system by using a 24-h stepwise addition of a 0.96 mM MB9 dye to immobilised cells containing the PpAzoR azoreductase under static conditions, which resulted in a slight increase in the final product yields (Table 2). Finally, one-pot reactions were set up using immobilised *E. coli* cells that have co-produced both the PpAzoR and the CotA enzymes, reducing twofold the time and cost associated with cell production (Table 2). One-pot bioprocesses show obvious advantages such as low costs, higher yields, and environmental benefits (Sydnes, 2014). The sequential activity of the two enzymes was controlled by the aeration conditions: first, static conditions during 24 h to promote PpAzoR activity, followed by stirring at 180 rpm for 24 h reaction, where CotA that couples the oxidation of substrates to the reduction of oxygen to water is active. This approach resulted in lower phenoxazinone yields (50%) as compared when using *E. coli* cells harbouring separately the target enzymes (Table 2). However, when the first step was performed in a recycled manner, i.e., through 24-h stepwise addition of dye solution, the overall performance improved significantly, affording 91% product yields. The activity recovery was measured in both the two-step and the one-pot reactions, and we found that after the first, second, and third cycles, the activity measured is around 100, 75, and 60% of the initial activity, respectively; it takes four cycles to observe a drop in the biocatalyst activity to values around 40% of the initial activity. The high yields of products achieved give confidence for their ease purification using preparative HPLC techniques. This enzymatic system resulted in 80–100% decolourisation of model acid, reactive, and direct dye baths (Mendes et al., 2011a). The toxicity after the sequential treatment was also significantly reduced, giving good indications that valuable compounds can be extracted from these model dye baths. Further studies to substantiate the biocatalytic efficiency of the system proposed using model and real wastewaters need to be performed in order to fully exploit the enzymatic bioprocesses described that respect principles of circularity, sustainability, and planetary boundaries.

**TABLE 2:** Bioconversion of MB9 using immobilised *Escherichia coli* KRX whole cells (120 mg DCW ml^–1^) overproducing each enzyme separately (two-step) and overproducing both enzymes simultaneously (one-pot).

Reaction	SAHBS (mM)	Yield step 1 (%)	Compound 7 (mM)	Yield step 2 (%)	Overall yield (%)
Two-step	0.71	74	0.36	99	74
	0.76	80	0.37	97	78
One-pot	–	–	0.24	–	50
	–	–	0.44	–	91

*The first step was performed in static conditions at 30°C upon addition of MB9 dye (1 cycle) or four 24-h sequential additions of MB9 dye (4 cycles). The second step was performed in stirring conditions, at 37°C, for 24 h. Reactions were performed in water.*

## Conclusion

Azo dyes are xenobiotic molecules whose structures were designed to resist decolourisation and degradation, which in turn challenge its sustainable and efficient eradication from the environment. We had proven that the sequential use of PpAzoR azoreductase and CotA-laccase enzymes resulted in the decolourisation, degradation, and conversion into valuable compounds of a set of azo dyes, mordant, acid, reactive and direct, and low-cost feedstock, commonly found in dye-containing wastewaters of textile-related industries. For the set-up of the biocatalytic bioprocess, we took advantage of the previously known complementary catalytic properties of azoreductases and laccases; i.e., azoreductases reduce azo dyes to aromatic amines and laccases promote their oxidative coupling into valuable aromatic compounds, precursors of biologically active molecules. Free and immobilised whole cells of *E. coli* containing these enzymes allowed developing an economically feasible bioprocess resulting in less contaminated reaction mixtures with final product yields that go up to **∼**90%. The optimised biocatalytic systems offer a sustainable and promising approach for cleaning up dye-containing wastewaters while producing valuable chemicals with a range of applications in chemical industries.

## Data Availability Statement

The raw data supporting the conclusions of this article will be made available by the authors, without undue reservation.

## Author Contributions

AF conducted most of the experiments. BP developed the two-step biotransformation system using purified enzymes. LB helped in developing whole-cell systems. MR made the NMR analysis. BR discussed the results and participated in the writing. LM proposed and supervised the whole project. All authors contributed to the article and approved the submitted version.

## Conflict of Interest

The authors declare that the research was conducted in the absence of any commercial or financial relationships that could be construed as a potential conflict of interest.
