# Supplementary material for: Wasteful Azo Dyes as a Source of Biologically Active Building Blocks
Source: Front Bioeng Biotechnol. 2021 Jun 15;9:672436. doi: 10.3389/fbioe.2021.672436 (PMC8239230; doi:10.3389/fbioe.2021.672436)
Supplement: Supplementary file 1 [file Data_Sheet_1.PDF]

Supplementary Material

Wasteful azo dyes as a source of biologically active building blocks

Ana Fernandes, Bruna Pinto, Lorenzo Bonardo, Beatriz Royo, M. Paula Robalo and Lígia O. Martins

[1]*Instituto de Tecnologia Química e Biológica António Xavier, Universidade Nova de Lisboa, Av da República, 2780-157 Oeiras, Portugal*

[2]*Área Departamental de Engenharia Química, ISEL - Instituto Superior de Engenharia de Lisboa, Instituto Politécnico de Lisboa, R. Conselheiro Emídio Navarro, 1, 1959-007 Lisboa, Portugal*

[3]*Centro de Química Estrutural, Complexo I; Instituto Superior Técnico, Universidade de Lisboa, Av. Rovisco Pais, 1049-001 Lisboa, Portugal*

Table S1 ^{1}H- and ^{13}C-NMR spectral data of products identified in this study.

**1**, SAHBS	^{1}H NMR (CD_3OD-d_4): δ 7.22 (d, J = 2.0 Hz, H2); 7.08 (dd, J=8.0, 2.0 Hz, H6); 6.72 (d, J=8.0, H5).
**2**, SAHNS	^{1}H NMR (CD_3OD-d_4): δ 8.71 (dd, J = 7.8, 1.2 Hz, 1H, H8), 7.93 (d, J = 7.8, 1.2 Hz, 1H, H5), 7.84 (s, 1H, H2), 7.38 (m, 2H, H6, H7). ^{13}C NMR (CD_3OD-d_4): δ 138.8 (C4), 132.3 (C4a), 131.3 (C1), 127.7 (C8), 126.1 (C7), 126.1 (C3), 125.8 (C8a), 124.7 (C6), 121.9 (C5), 118.9 (C2).
**3**, DAHNS	^{1}H NMR (CD_3OD-d_4): δ 7.95 (d, J = 1.2 Hz, 1H, H4), 7.69 (d, J = 8.8 Hz, 1H, H5), 7.65 (d, J = 8.8 Hz, 1H, H6), 7.37 (d, J = 1.2 Hz, 1H, H2).<br>^{13}C NMR (CD3OD-d4): 152.9 (C1), 141.1 (C3), 130.6 (C8), 130.4 (C7), 122.7 (C5), 117.9 (C6), 116.6 (C4), 114.2 (C4a), 113.9 (C8a), 105.2 (C2).
**4**, SANT	^{1}H NMR (CD_3OD-d_4): δ 8.53 (s, 1H, H2), 8.34 (s, 1H, H4), 8.27 (s, 1H, H5), 7.98 (s, 1H, H8).
**5**, SABS	^{1}H NMR (CD_3OD-d_4): δ 7.55 (d, J= 8.8 Hz, 2H, H3, H5), 6.68 (d, J= 8.8 Hz, 2H, H2, H6).
**6**, SDBS	^{1}H NMR (CD_3OD-d_4): δ 7.19 (d, J = 2.8 Hz, 1H, H6); 6.72 (dd, J = 8.4 Hz, 2.4 Hz, 1H, H4); 6.67 (d, J = 8.4 Hz, 1H, H3).<br>^{13}C NMR (CD_3OD-d_4): 137.4 (C2), 136.8 (C5), 129.3 (C1), 120.2 (C4), 118.6 (C3), 115.0 (C6).
**7**	^{1}H NMR (CD_3OD-d_4) (Forte et al., 2010): δ 8.14 (d, J= 2.0, H9); 7.89 (dd, J = 8.4; 2.0 Hz, H7); 7.52 (d, J=8.4 Hz, H6); 6.49 (s, H5); 6.43 (s, H8).
**8**	^{1}H NMR (CD_3OD-d_4) (Sousa et al, 2020): δ 8.76 (dd, J = 8.4 , 1.0 Hz, 1H, H8), 8.15 (dd, J = 7.8 , 1.5 Hz, 1H, H5), 7.61 (dt, J = 8.0 , 1.5 Hz, 1H, H6), 7.51 (dt, J = 8.4 , 1.0 Hz, 1H, H7), 6.91 (s, 1H, H2). ^{13}C NMR (D_2O)[a]: 183.5 (C4), 180.1 (C3), 155.2 (C1), 137.0 (C7), 132.2 (C6), 131.8 (C4a), 131.5 (C5), 130.9 (C8a), 130.2 (C2), 126.5 (C8).
**9**	^{1}H NMR (D_2O) (Sousa et al., 2014)[a]: δ 7.04 (d, 2H, J = 9.5 Hz, H4, H9); 6.38 (d, 2H, J = 9.5 Hz, H3, H8).^{13}C NMR (D_2O)[a]: δ 146.6 (C2, C7); 139.5 (C1a, C5a); 137.9 (C4a, C9a); 132.9 (C4, C9); 127.2 (C3, C8); 111.3 (C1, C6).

[a] referenced to $(CD_3)_2CO$-d_6 solvent.

Table S2 Expected products of azo dyes degradation by *P. putida* MET94 PpAzoR azoreductase accordingly to the reductive enzymatic mechanism.

**Azo Dye**	**Aromatic amines**
**Mordant black 9** 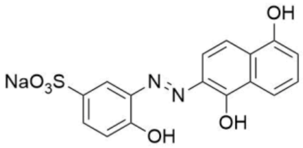	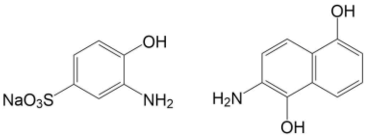 **1**
**Mordant black 3** 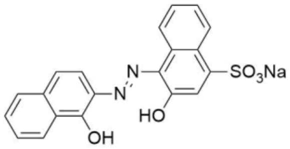	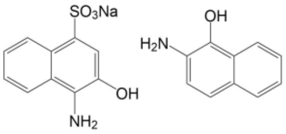 **2**
**Acid red 266** 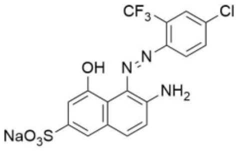	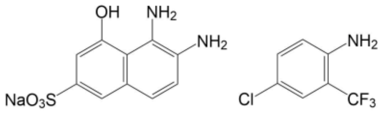 **3**
**Reactive yellow 145** 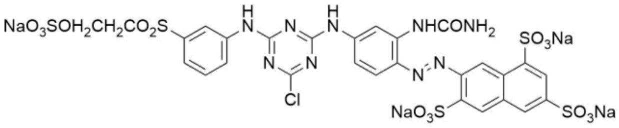	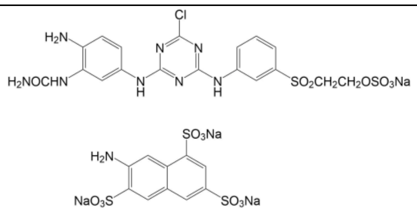 **4**
**Direct red 80** 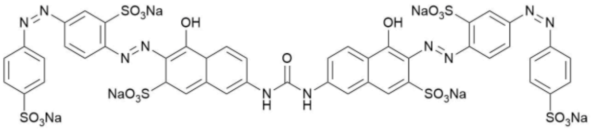	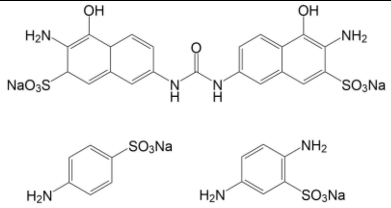 **5** **6**

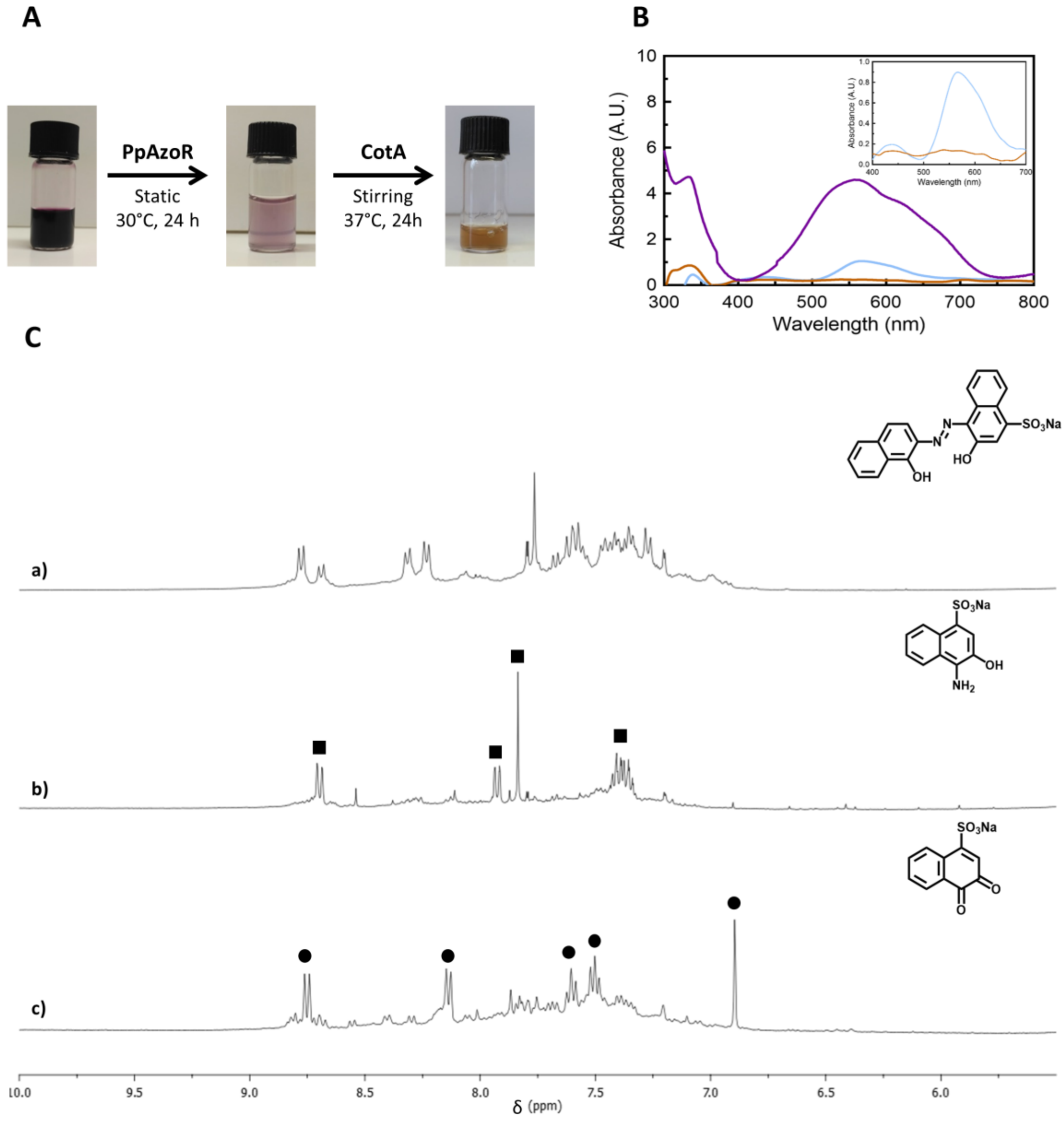

Figure S1. (A) Two-step sequential bioconversion of MB3 using purified PpAzoR azoreductase and CotA-laccase, under anaerobic and aerobic conditions, respectively. (B) UV-Vis spectra of initial reaction mixture (purple), after the PpAzoR azoreductase (light blue) and CotA-laccase (light brown) reactions (insert: zoom for lower absorbances). (C) ^{1}H-NMR spectra (aromatic region) of MB3 (a), and products of reaction after addition of PpAzoR azoreductase (b) and CotA-laccase (c). Resonances due to SAHNS (**2**) (filled squares) and orto-naphtoquinone (**8**) (filled circles) are labelled.

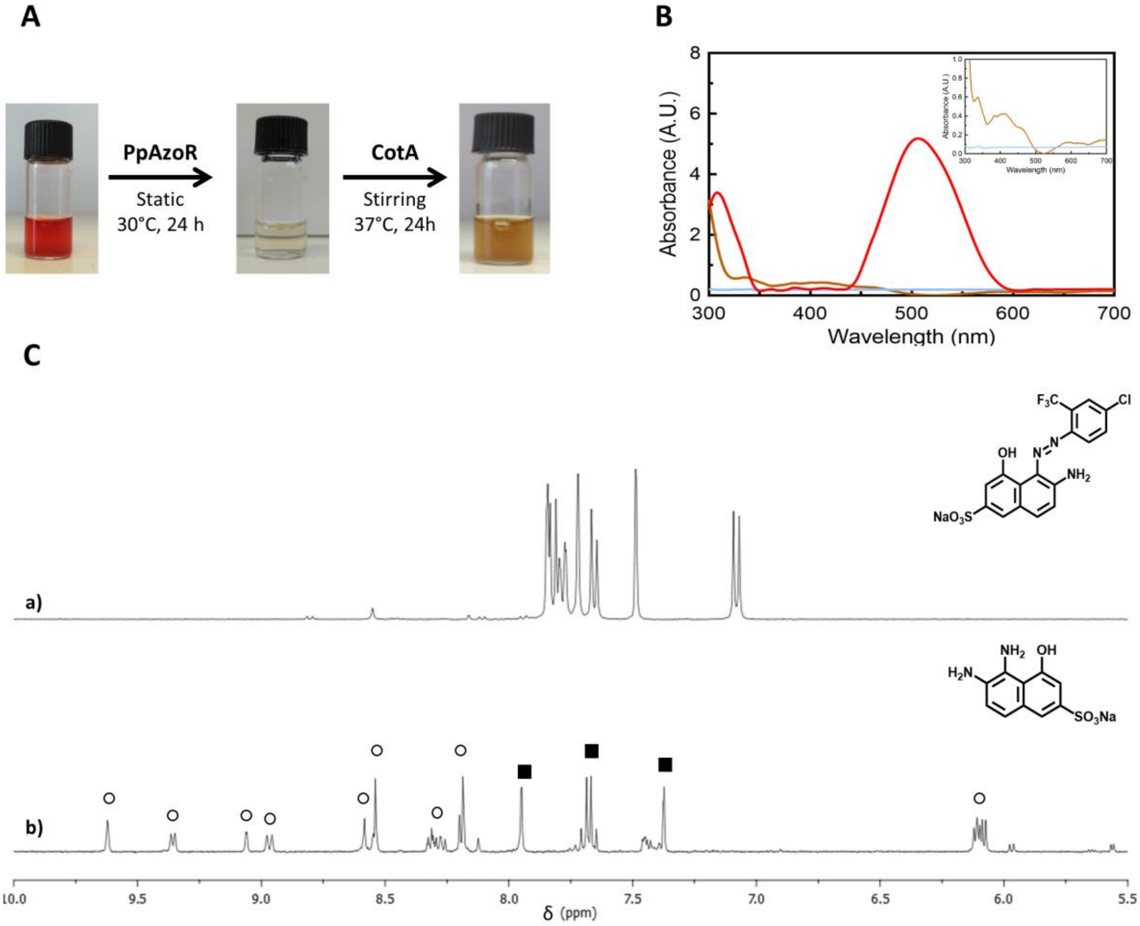

Figure S2 (A) Two-step bioconversion of AR266 with purified PpAzoR azoreductase and CotA-laccase, under anaerobic and aerobic conditions, respectively. (B) UV-Vis spectra of initial reaction mixture (dark orange), after the PpAzoR azoreductase (light blue) and CotA-laccase (light brown) reactions (insert: zoom for lower absorbances). (C) ^{1}H-NMR spectra (aromatic region) of AR266 (a), and products of reaction of PpAzoR azoreductase (b). Resonances due to NAD^+ and other intermediates resulting from NADH/NAD^+ degradation (open circles), and SDAHNS (**3**) (filled squares).

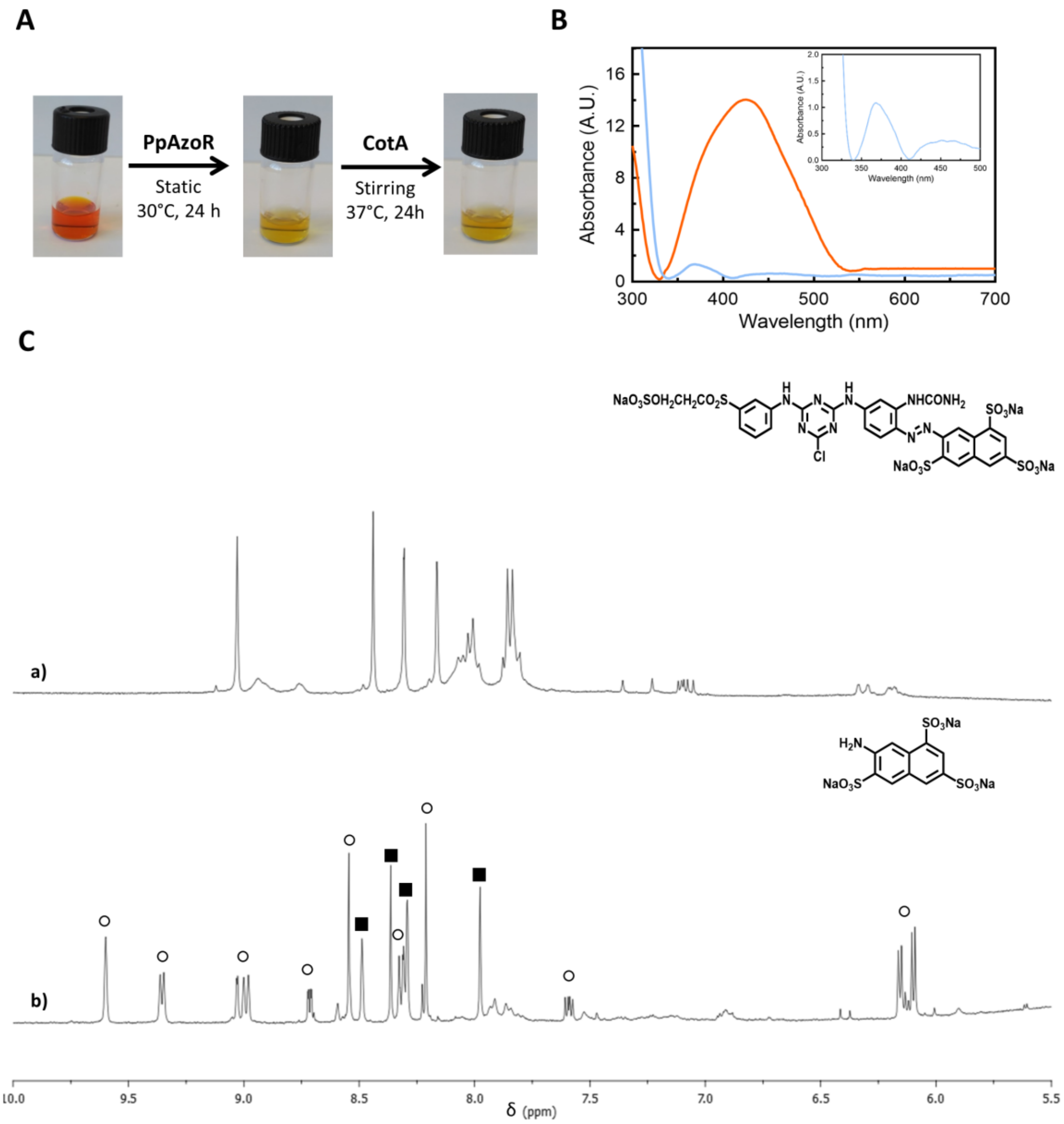

Figure S3 (A) Two-step bioconversion of RY145 bioconversion with purified PpAzoR azoreductase and CotA-laccase, under anaerobic and aerobic conditions, respectively. (B) UV-Vis spectra of initial reaction mixture (orange) and after the PpAzoR azoreductase reaction (or after CotA) (light blue) (insert: zoom for lower absorbances). (C) ^{1}H-NMR spectra (aromatic region) of RY145 (a), and products of reaction with PpAzoR azoreductase (similar to spectra after addition of CotA) (b). Resonances due to NAD^+ and other intermediates resulting from NADH/NAD^+ degradation (open circles), and SANT (**4**) (filled squares) are labelled.

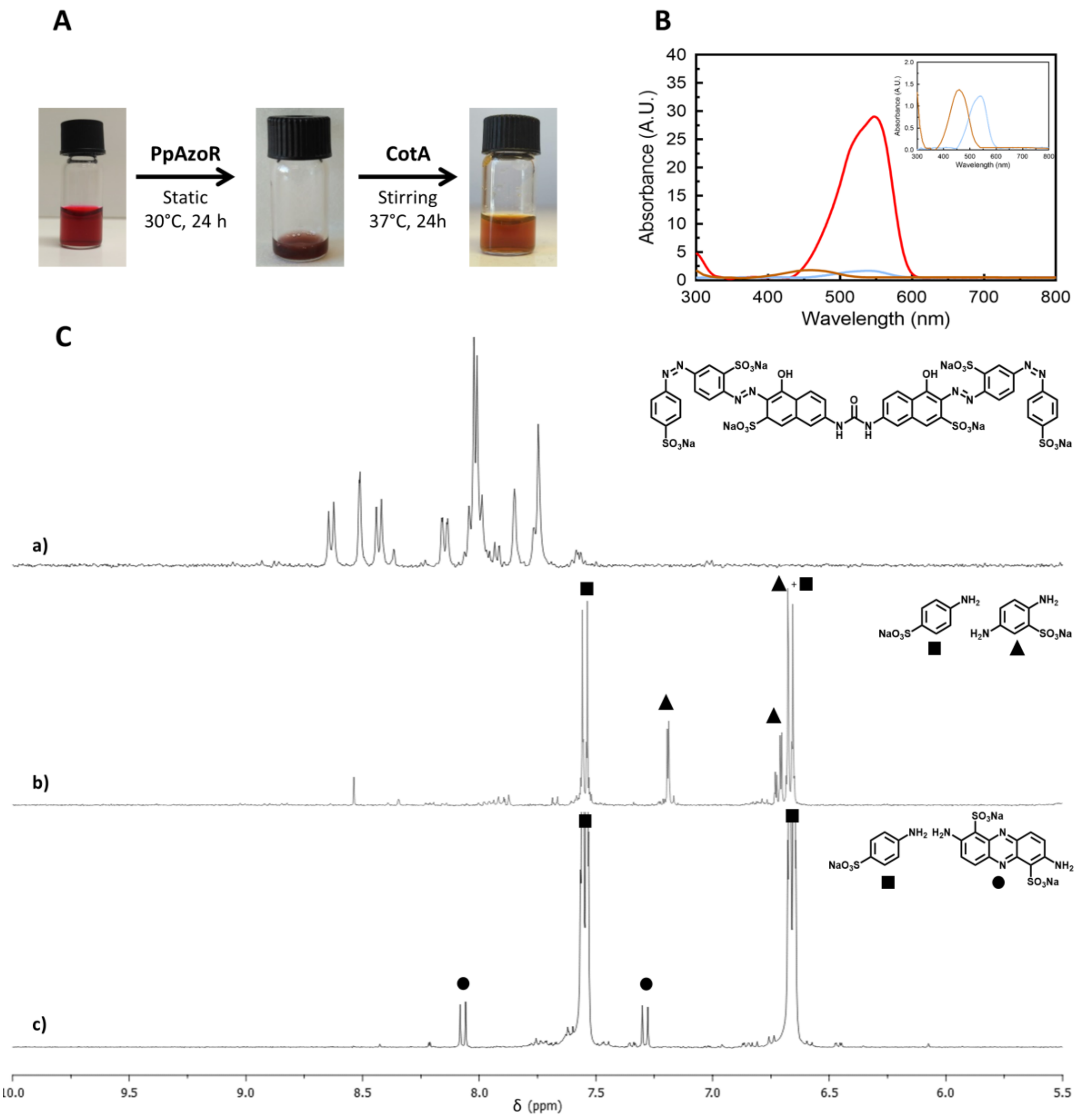

Figure S4 (A) Two-step bioconversion of DR80 with purified PpAzoR azoreductase and CotA-laccase, under anaerobic and aerobic conditions, respectively. (B) UV-Vis spectra of initial reaction mixture (dark orange), after the PpAzoR azoreductase (light blue) and CotA-laccase (light brown) reactions. (C) ^{1}H-NMR spectra (aromatic region) of DR80 (a), and the products of reaction of PpAzoR azoreductase (b) and CotA-laccase (c). Resonances due to SABS (**5**) (solid squares), SDBS (**6**) (solid triangles) and phenazine (**9**) (solid circles) are labelled.

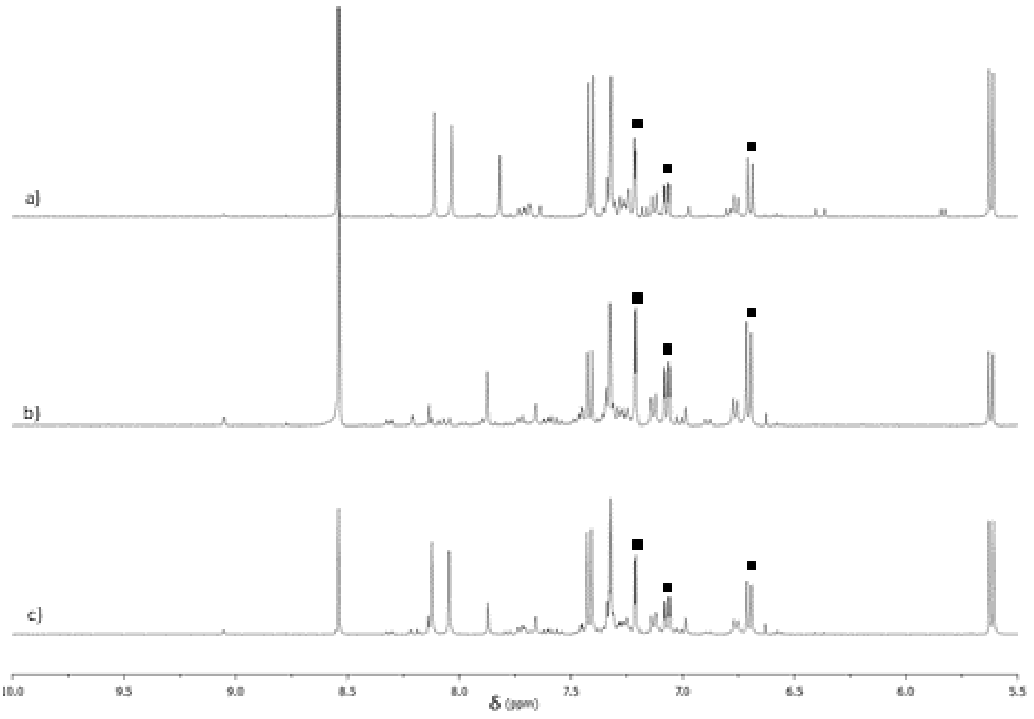

Figure S5 ^{1}H NMR spectra (aromatic region) of MB9 bioconversion using (a) *E. coli* Tuner, (b) *E. coli* KRX or (c) *E. coli* BL21 star cells containing PpAzoR azoreductase, after 72 h of reaction at 30°C, in static conditions. Resonances due to sodium SABHS (**1**) are labelled as filled squares.

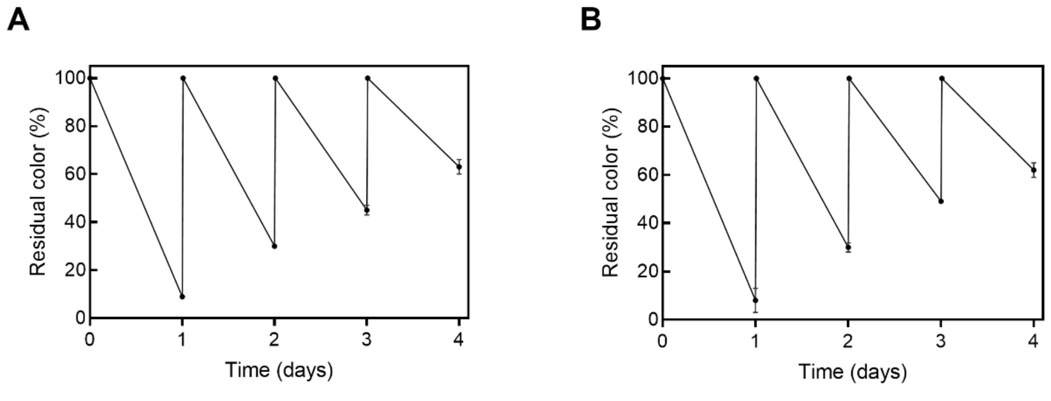

Figure S6 Whole-cell biocatalysis using alginate immobilized *E.coli* cells containing recombinant PpAzoR. Color in reaction mixtures in water (A) and 20 mM sodium phosphate buffer, pH 7 (B) after the 24 h-stepwise addition of 2 mM MB9.
